# Supplementary material for: Laminar Specificity of the Auditory Perceptual Awareness Negativity: A Biophysical Modeling Study
Source: bioRxiv. 2023 Mar 8:2023.03.06.531459. Preprint. [Version 1] doi: 10.1101/2023.03.06.531459 (PMC10028885; doi:10.1101/2023.03.06.531459)
Supplement: Supplement 1 [file NIHPP2023.03.06.531459v1-supplement-1.pdf]

bioRxiv preprint doi: https://doi.org/10.1101/2023.03.06.531459; this version posted March 8, 2023. The copyright holder for this preprint (which was not certified by peer review) is the author/funder, who has granted bioRxiv a license to display the preprint in perpetuity. It is made available under aCC-BY 4.0 International license.

Supplemental Figures

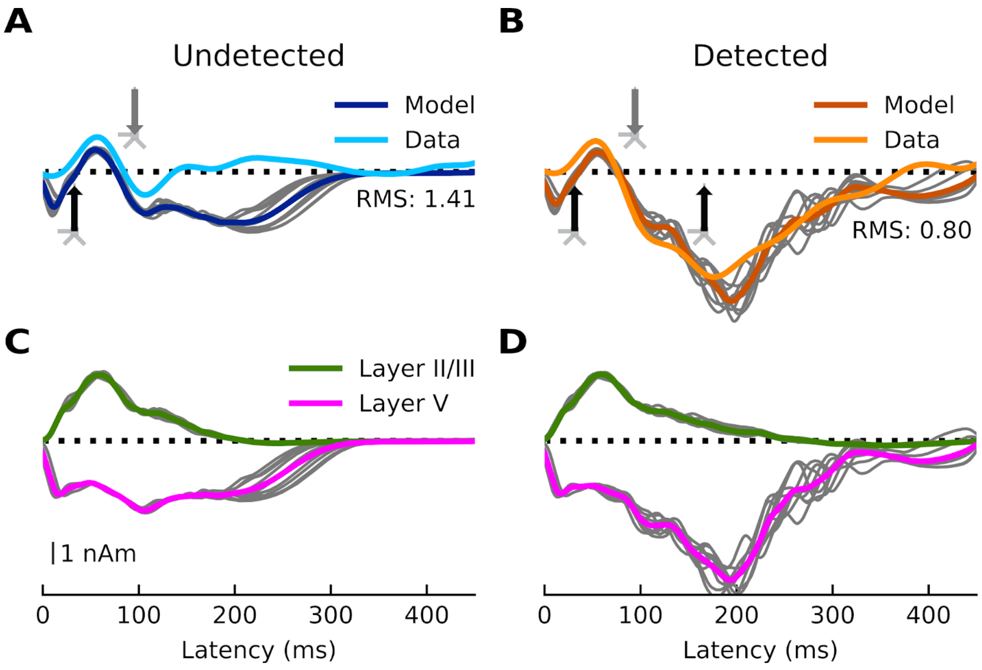

Fig S1. Current dipoles and laminar profiles for the perisomatic inhibition model. (**A**) Model output (dark blue) and data (light blue) for undetected target tones. A proximal input (36 ms) followed by a distal input (84.3 ms) drive the network. RMSE between empirical and simulated data is 1.41. (**B**) Model output (dark orange) and data (light orange) for detected target tones. The same proximal and distal inputs used to model the response to undetected target tones, and an additional proximal input (169.3 ms), drive the network. RMSE between empirical and simulated data is 0.80. (**C-D**) Laminar profiles for the responses to undetected and detected target tones. The corresponding input parameter values are displayed in Fig S4. The network from which the simulated dipole activity arises consists of 60,000 cells.

bioRxiv preprint doi: https://doi.org/10.1101/2023.03.06.531459; this version posted March 8, 2023. The copyright holder for this preprint (which was not certified by peer review) is the author/funder, who has granted bioRxiv a license to display the preprint in perpetuity. It is made available under aCC-BY 4.0 International license.

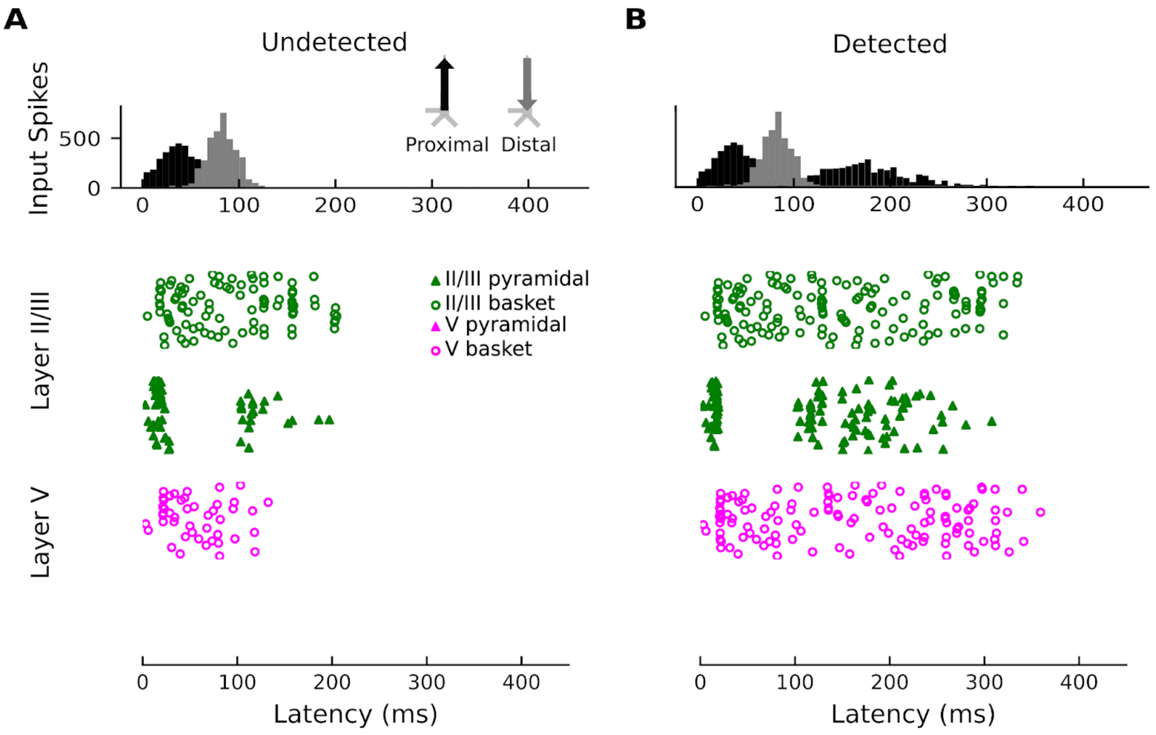

Fig S2. Spiking cell activity for the perisomatic inhibition model. (**A**) Network spiking activity for undetected target tones. (**B**) Network spiking activity for detected target tones. Note in both cases the complete absence of spiking activity in layer-V PNs.

bioRxiv preprint doi: https://doi.org/10.1101/2023.03.06.531459; this version posted March 8, 2023. The copyright holder for this preprint (which was not certified by peer review) is the author/funder, who has granted bioRxiv a license to display the preprint in perpetuity. It is made available under aCC-BY 4.0 International license.

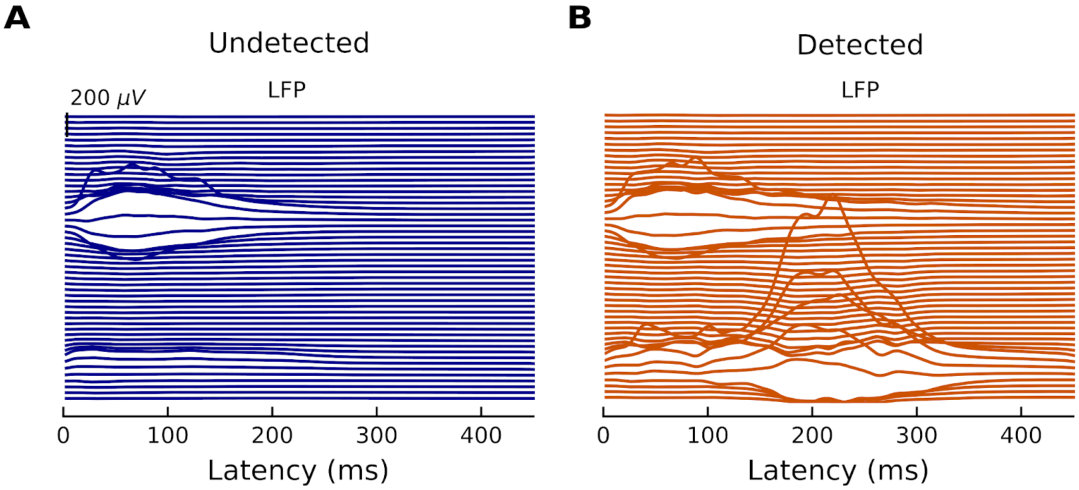

Fig S3. Local field potentials for the perisomatic inhibition model. (**A**) Simulated LFP to undetected target tones. (**B**) Simulated LFP to detected target tones.

bioRxiv preprint doi: https://doi.org/10.1101/2023.03.06.531459; this version posted March 8, 2023. The copyright holder for this preprint (which was not certified by peer review) is the author/funder, who has granted bioRxiv a license to display the preprint in perpetuity. It is made available under aCC-BY 4.0 International license.

Parameter			Undetected		Detected		
			Proximal	Distal	Proximal	Distal	Proximal
Input time (ms)			36	84.275	36	84.275	169.273
SD (ms)			25	15.063	25	15.063	50.396
Weight (µS)	Layer II/III Pyramidal	AMPA	2.2	0.005	2.2	0.005	0.364
		NMDA	3.2	0.015	3.2	0.015	0
	Layer II/III Basket	AMPA	2.028	0.0645	2.028	0.0645	2
		NMDA	0.916	0.0988	0.916	0.0988	2
	Layer V Pyramidal	AMPA	0.001	0.00025	0.001	0.00025	0.005
		NMDA	0.000999	0.00745	0.000999	0.00745	0.994
	Layer V Basket	AMPA	0.4751		0.4751		2
		NMDA	0.0614		0.0614		2

Fig S4. Input parameters for the perisomatic inhibition model. The canonical circuit model was modified by increasing the conductance of the $GABA_B$ receptors on the perisomatic compartment of layer-V PNs.

bioRxiv preprint doi: https://doi.org/10.1101/2023.03.06.531459; this version posted March 8, 2023. The copyright holder for this preprint (which was not certified by peer review) is the author/funder, who has granted bioRxiv a license to display the preprint in perpetuity. It is made available under aCC-BY 4.0 International license.

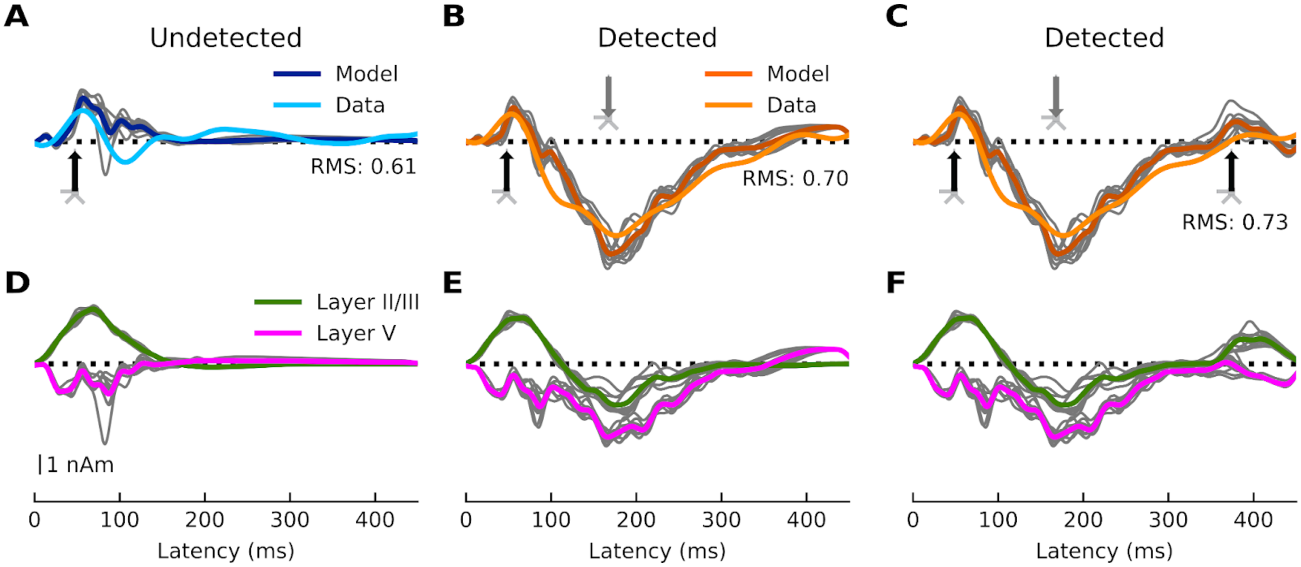

Fig S5. Current dipoles and laminar profiles for the reduced-input (A, B, D, E) and proximal-distal-proximal (C, F) input sequences. (**A**) Model output (dark blue) and data (light blue) for undetected target tones. A single proximal input (47.8 ms) drives the network. RMSE between empirical and simulated data is 0.61. (**B**) Model output (dark orange) and data (light orange) for detected target tones for the reduced input sequence model. The same proximal input that was used to model the response to undetected target tones, and a distal input (154.0 ms) drive the network. RMSE between empirical and simulated data is 0.70. (**C**) Model output (dark orange) and data (light orange) for detected target tones for the proximal-distal-proximal model. The same proximal input that was used to model the response to undetected target tones, a distal input (154.0 ms), and an additional proximal input (395.4 ms) drive the network. RMSE between empirical and simulated data is 0.73. (**D-F**) Laminar profiles for the responses to the undetected and detected target tones. The corresponding input parameter values are displayed in Fig S8 and S9. The network from which the simulated dipole activity arises consists of 60,000 cells.

bioRxiv preprint doi: https://doi.org/10.1101/2023.03.06.531459; this version posted March 8, 2023. The copyright holder for this preprint (which was not certified by peer review) is the author/funder, who has granted bioRxiv a license to display the preprint in perpetuity. It is made available under aCC-BY 4.0 International license.

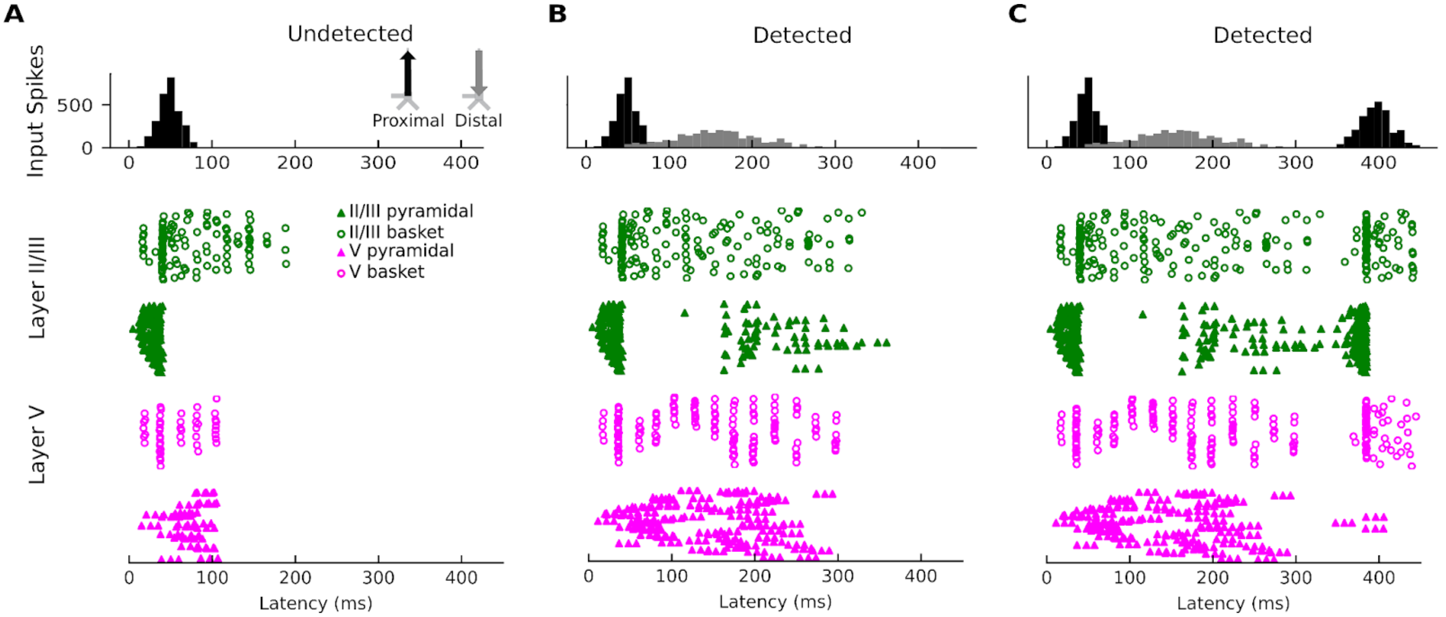

Fig S6. Spiking cell activity for the reduced-input and proximal-distal-proximal sequences. (**A**) Network spiking activity for undetected target tones. (**B**) Network spiking activity for detected target tones for the reduced input sequence. (**C**) Network spiking activity for the detected target tones for the proximal-distal-proximal input sequence.

bioRxiv preprint doi: https://doi.org/10.1101/2023.03.06.531459; this version posted March 8, 2023. The copyright holder for this preprint (which was not certified by peer review) is the author/funder, who has granted bioRxiv a license to display the preprint in perpetuity. It is made available under aCC-BY 4.0 International license.

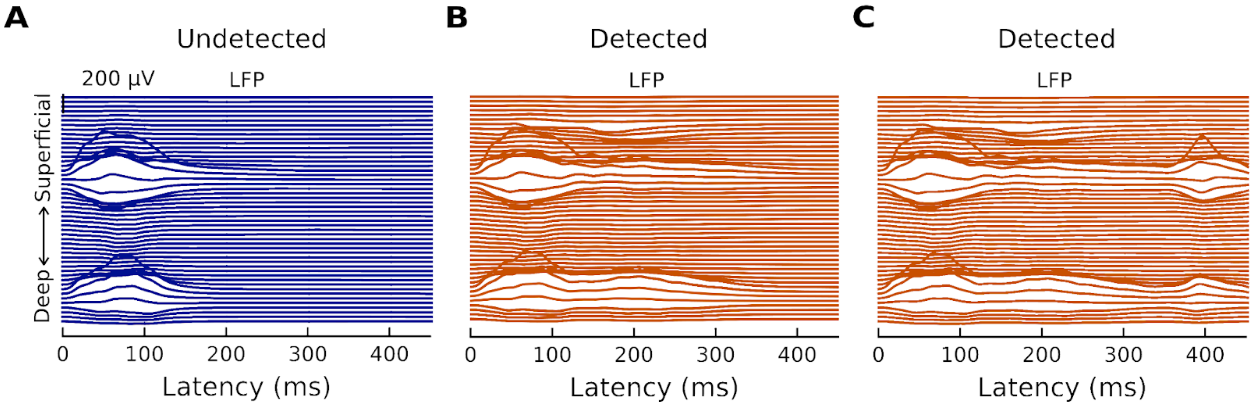

Fig S7. Local field potentials for the reduced-input and proximal-distal-proximal sequences. (**A**) Simulated LFP for undetected target tones. (**B**) Simulated LFP for detected target tones for the reduced input sequence. (**C**) Simulated LFP for detected target tones for the proximal-distal-proximal input sequence.

bioRxiv preprint doi: https://doi.org/10.1101/2023.03.06.531459; this version posted March 8, 2023. The copyright holder for this preprint (which was not certified by peer review) is the author/funder, who has granted bioRxiv a license to display the preprint in perpetuity. It is made available under aCC-BY 4.0 International license.

Parameter			Undetected	Detected	
			Proximal	Proximal	Distal
			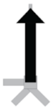	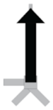	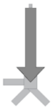
Input time (ms)			47.815	47.815	153.95
SD (ms)			13.317	13.317	55.1
Weight (µS)	Layer II/III Pyramidal 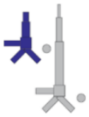	AMPA	0.659	0.659	0.607
		NMDA	0.535	0.535	0.443
	Layer II/III Basket 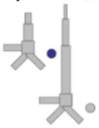	AMPA	1.097	1.097	0.623
		NMDA	0.987	0.987	1.059
	Layer V Pyramidal 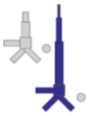	AMPA	0.004	0.004	0.6
		NMDA	0.00903	0.00903	0.3
	Layer V Basket 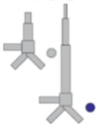	AMPA	0	0	
		NMDA	0	0	

Fig S8. Input parameters for the reduced input sequence. The model column used was the calcium model column that consists of a more biologically accurate distribution of Ca^{2+} channels on layer-V PNs compared to HNN's original Jones 2007 model column.

bioRxiv preprint doi: https://doi.org/10.1101/2023.03.06.531459; this version posted March 8, 2023. The copyright holder for this preprint (which was not certified by peer review) is the author/funder, who has granted bioRxiv a license to display the preprint in perpetuity. It is made available under aCC-BY 4.0 International license.

**Parameter**			**Undetected**	**Detected**		
			**Proximal**	**Proximal**	**Distal**	**Proximal**
			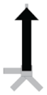	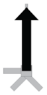	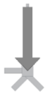	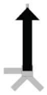
**Input time (ms)**			47.815	47.815	153.95	395.389
**SD (ms)**			13.317	13.317	55.1	20
**Weight (µS)**	**Layer II/III Pyramidal**	**AMPA**	0.659	0.659	0.607	0.364
	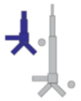	**NMDA**	0.535	0.535	0.443	0.0269
	**Layer II/III Basket**	**AMPA**	1.097	1.097	0.623	0.995
	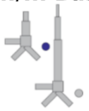	**NMDA**	0.987	0.987	1.059	0.993
	**Layer V Pyramidal**	**AMPA**	0.004	0.004	0.6	0.00544
	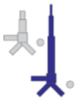	**NMDA**	0.00903	0.00903	0.3	0
	**Layer V Basket**	**AMPA**	0	0		0.984
	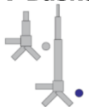	**NMDA**	0	0		0.998

Fig S9. Input parameters for the proximal-distal-proximal input sequence. The model column used was the calcium model column that consists of a more biologically accurate distribution of Ca^{2+} channels on layer-V PNs compared to HNN's original Jones 2007 model column.
